# Supplementary figures and images for: Legionella pneumophila exploits the endo-lysosomal network for phagosome biogenesis by co-opting SUMOylated Rab7
Source: PLoS Pathog. 2024 May 13;20(5):e1011783. doi: 10.1371/journal.ppat.1011783 (PMC11115209; doi:10.1371/journal.ppat.1011783)

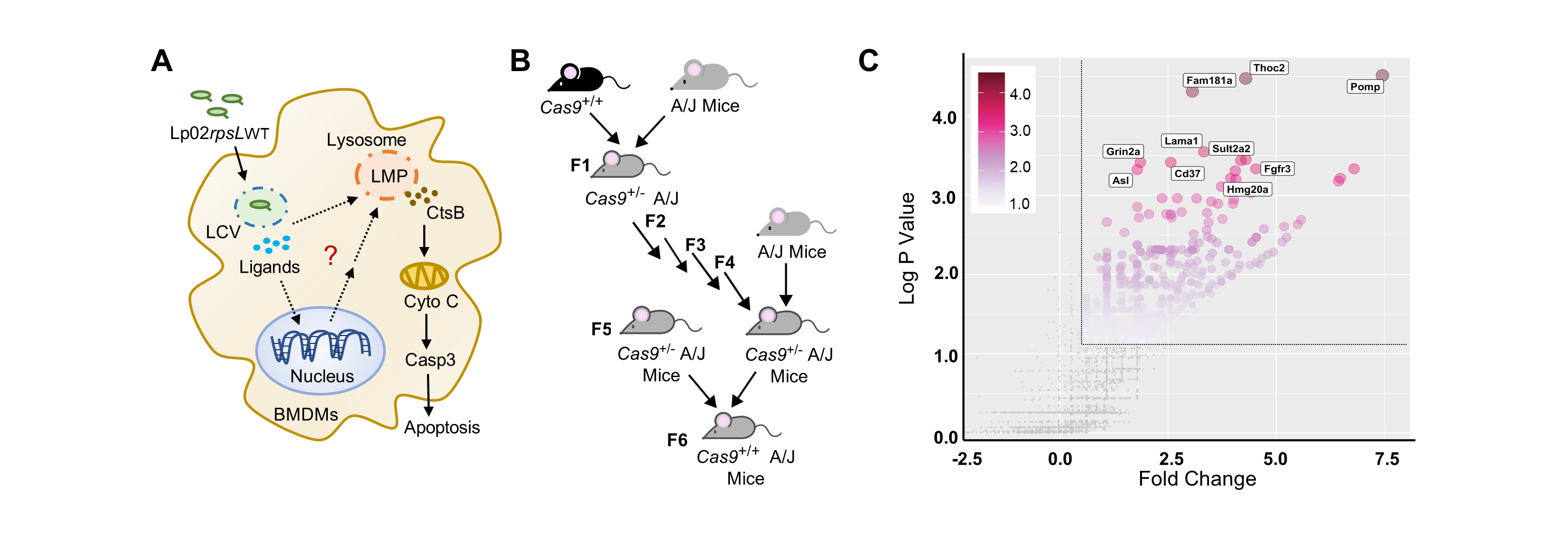

Supplement: S1 Fig — (A) Model of the lysosomal macrophage death triggered by infections with strain Lp02rpsLWT. Ligands released by the bacterium trigger lysosomal membrane permeabilization and apoptosis in BMDMs, resulting in the termination of bacterial replication. (B) Generation of Cas9+/+ mice with A/J background. Cas9+/+ mice with B6 background were breeding with A/J mice, their off-springs carrying heterozygous Cas9+/- gene were backcrossed with A/J mice for an additional three generations. Cas9+/+ mice with A/J background were generated by mating between Cas9+/- A/J mice. (C) Volcano plot of significant genes from samples containing large LCVs vs those containing small LCVs during CRISPR/Cas9 knockout screenings. Threshold: Fold change > 1.5, -log10 p value > 1, (P value <0.1). The 10 genes with the highest changes were highlighted. (TIF) [file ppat.1011783.s001.tif]

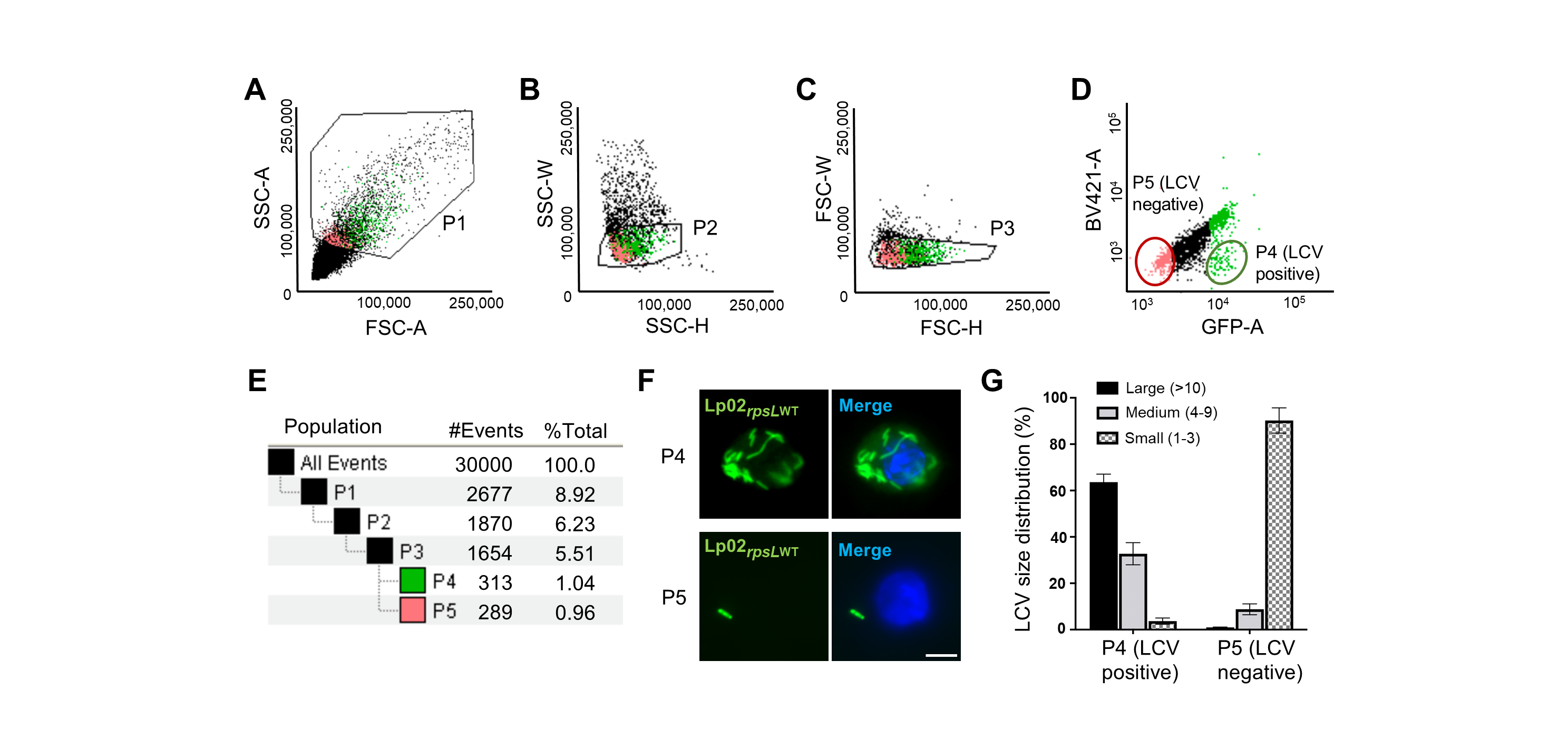

Supplement: S2 Fig — (A) Forward scatter vs size scatter. Samples with estimated mouse macrophage size, complexity and infected by strain Lp02rpsLWT(pGFP) were collected. Cell debris and dead cells were excluded from the sorting flow. (B) Size scatter heights vs weights. Single macrophages were collected. Aggregated cells were excluded from sorting flow. (C) Forward scatter heights vs weights. Cells stacked together were excluded from the sorting flow. (D) GFP signal vs background signal. BV421 was set as the background control to separate the signal away from macrophage autofluorescence. P4 represents macrophages containing large LCVs while P5 represents macrophages containing only one bacterium. (E) Percentage of cells in each portion. P4 or P5 accounts for 1% of total cell samples being examined. (F) Representative images of macrophages containing large LCVs in P4 or small LCVs in P5 sorted by flow cytometry. DNA was stained with Hoechst. Bar, 5 μm. (G) LCV size distribution of macrophages in P4 and P5 portions. At least 300 LCVs were scored for each sample. (TIF) [file ppat.1011783.s002.tif]

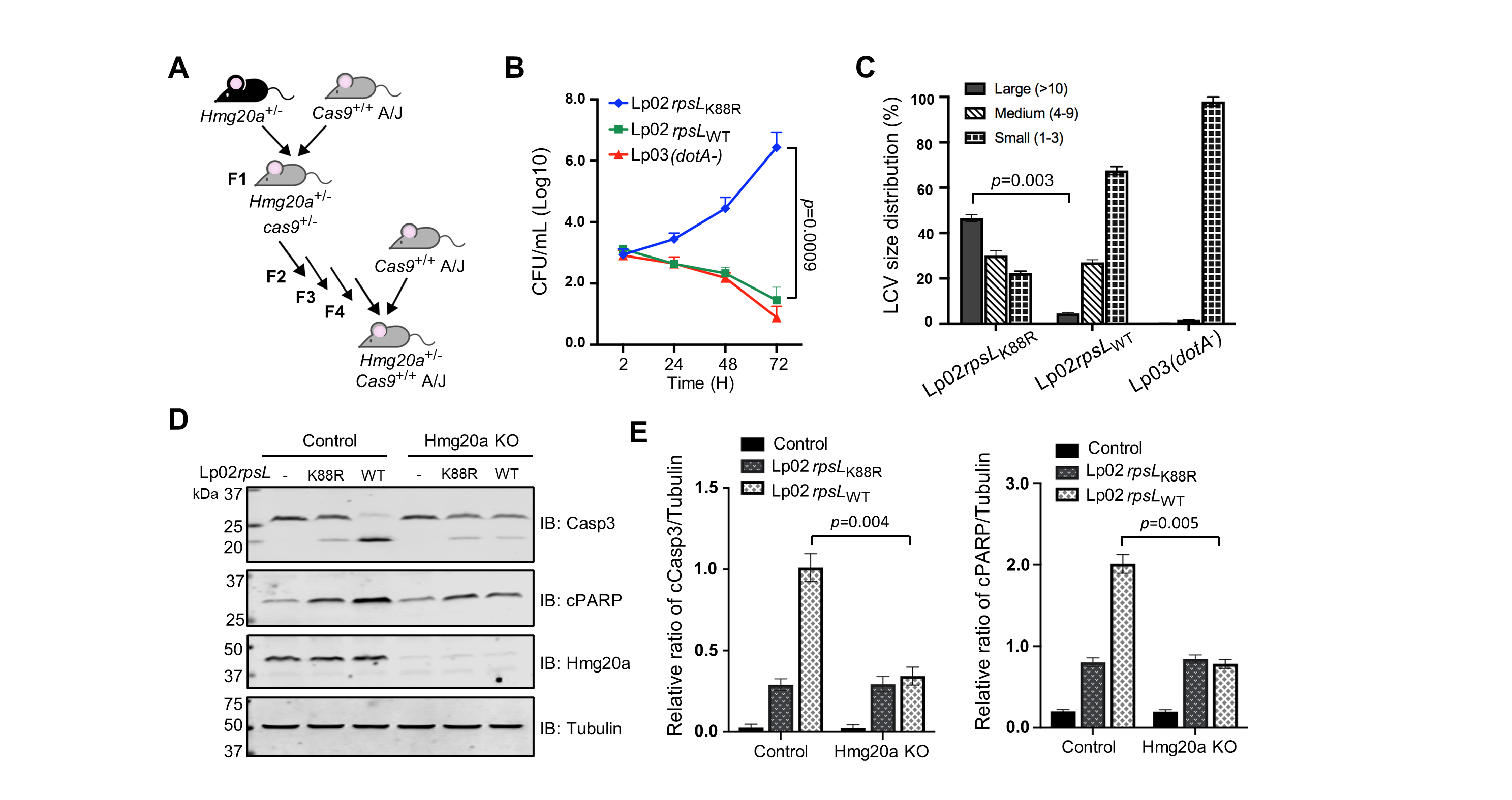

Supplement: S3 Fig — (A) Hmg20a+/- mice in B6 background were bred with Cas9+/+ A/J mice, and their off-springs with the Hmg20a+/- Cas9+/- genotype were bred with Cas9+/+ A/J mice for an additional four generations. Hmg20a+/- Cas9+/+ mice with A/J background were generated by mating Hmg20a+/- Cas9+/- A/J mice with Cas9+/+ A/J mice. (B) Intracellular replication of relevant L. penumophila strains in BMDMs from Hmg20a+/- Cas9+/+ A/J mice. BMDMs were infected with bacteria at an MOI of 0.05. Samples were plated at indicated time points to obtain total colony-forming units. Data represents mean ± s.e. from three experiments each done in triplicate. (C) The distribution LCVs of different sizes formed by the indicated L. penumophila strains in BMDMs from Hmg20a+/- Cas9+/+ A/J mice. BMDMs were infected by indicated strains at an MOI of 1 and infection were proceeded for 14 h before fixation and analysis. (D) Protein levels of apoptosis-associated proteins in Hmg20a knockout BMDMs infected by relevant L. penumophila strains. Cells were lysed 8 hpi and processed for immunoblotting using antibodies specific for caspase3, cleaved RARP, Hmg20a or Tubulin. (E) The relative ratio of the band intensity of cleaved caspase3, cleaved RARP to Tubulin. Date shown as mean ± s.e. of relative ratio calculated from three independent experiments. (TIF) [file ppat.1011783.s003.tif]

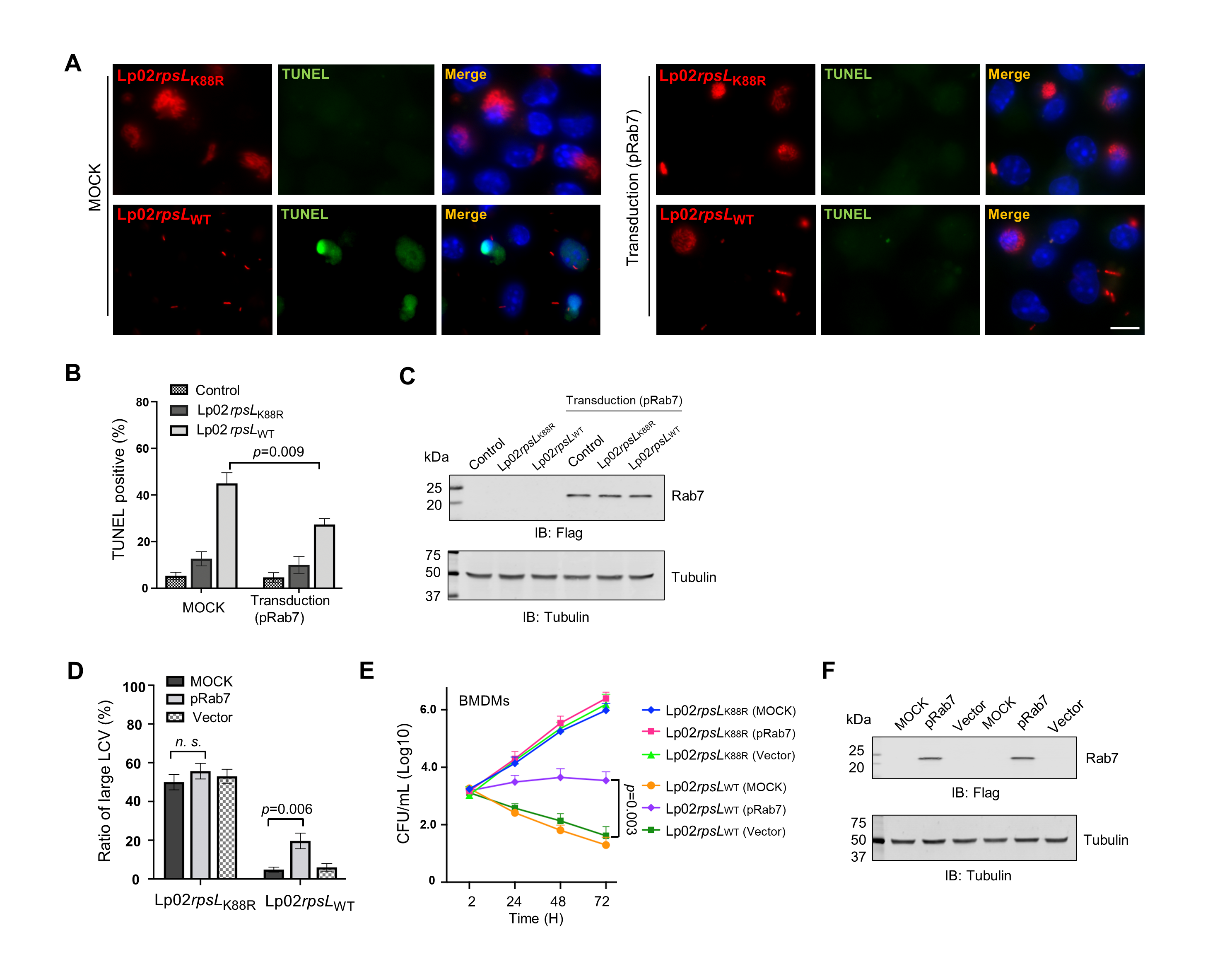

Supplement: S4 Fig — (A-C) Cell death was measured by TUNEL staining. Images are representatives of one of the three experiments with similar results. Bar, 10 μm (A). Quantitative results (mean ± s.e.) were from three independent experiments each done in triplicate (B). Expression of Rab7 in BMDMs were detacted by immunoblotting (C). (D-F) Overexpression of Rab7 did not detectably impact intracellular growth of strain Lp02rpsLK88R in BMDMs. The indicated bacterial strains were used to infect BMDMs, the ratio of large LCVs (D) and total bacterial counts for a 72-h experimental duration at 24-h intervals (E) were analyzed. In all cases, quantitative results (mean ± s.e.) were from three independent experiments each. Expression of Rab7 in BMDMs were detacted by immunoblotting (F). (TIF) [file ppat.1011783.s004.tif]

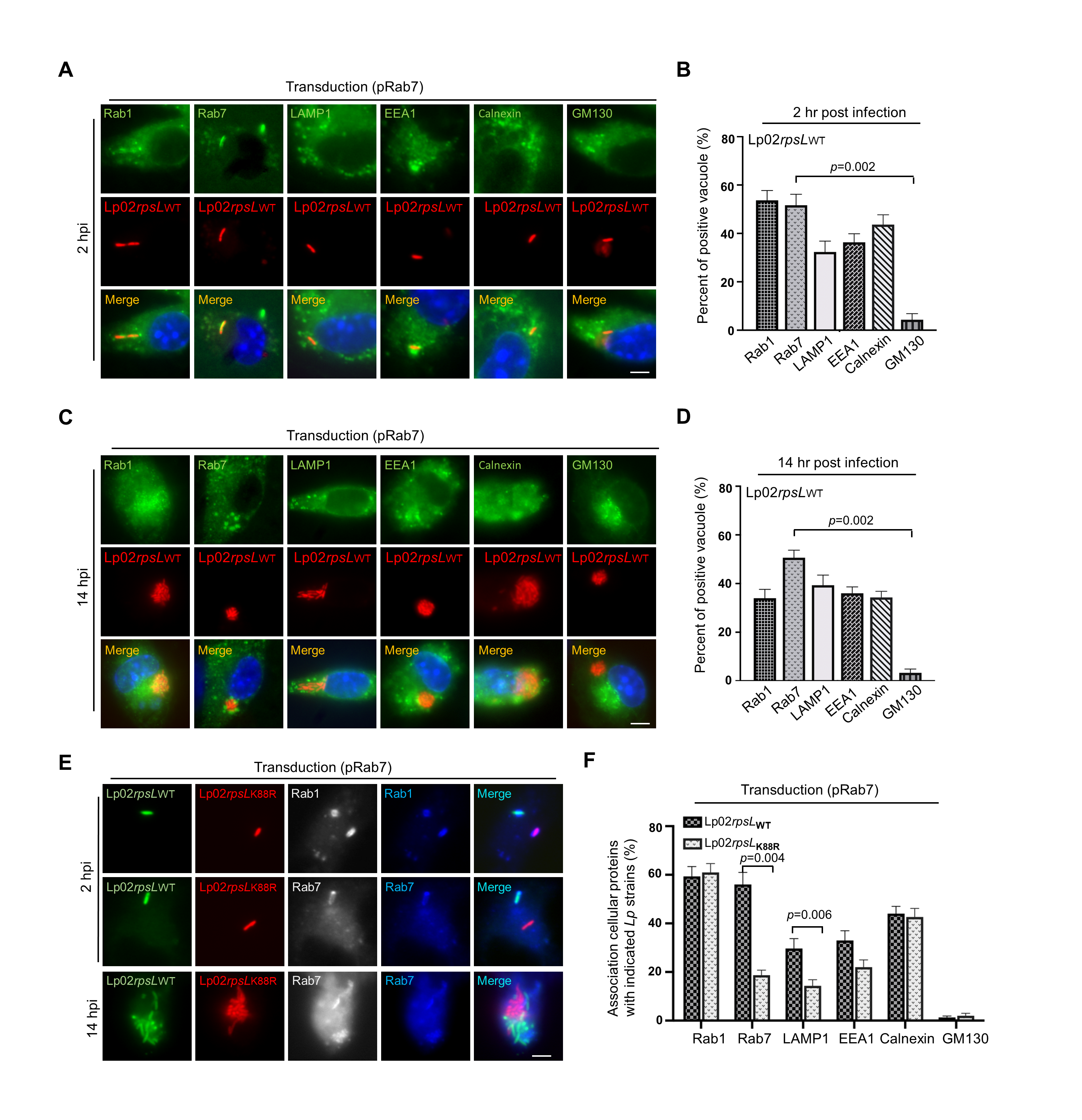

Supplement: S5 Fig — (A-D) Representative images of LCVs associated with cellular organelles or relevant proteins in BMDMs infected by strain Lp02rpsLWT for 2 h (A) or 14 h (C). BMDMs were transduced by lentiviruses that direct Rab7 expression. 2 d post transduction, cells were infected by bacteria at an MOI of 1. Bar, 5 μm. Quantitation of the association of the indicated proteins with LCVs at 2 hpi (B) or 14 hpi (D). At least 300 LCVs were counted for each sample. (E) The distribution of Rab7 in BMDMs co-infected with strains Lp02rpsLWT and Lp02rpsLK88R. Note that Rab7 signals on LCVs formed by strain Lp02rpsLWT were stronger than those on strain Lp02rpsLK88R. Bar, 5 μm. (F) Quantitation of the association of relevant proteins with LCVs in BMDMs. Results (mean ± s.e.) were collected from three independent experiments. (TIF) [file ppat.1011783.s005.tif]

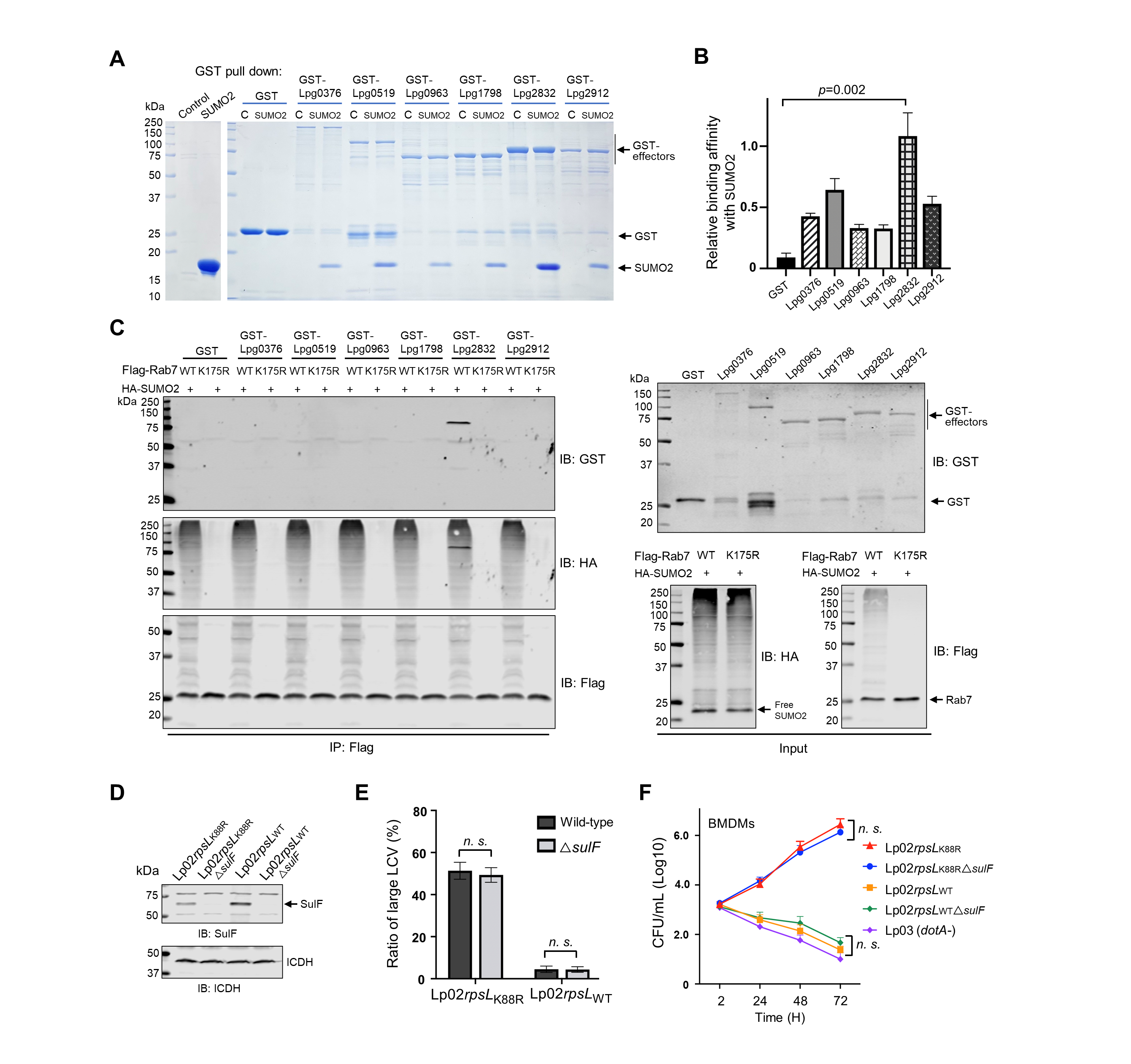

Supplement: S6 Fig — (A) Evaluation of the binding by GST pulldown assays. Lysates of E. coli expressing the candidate proteins were incubated with GST resins (5%) for 2 h. Washed GST resins coated with candidate effectors were incubated with purified SUMO2 for another 1 h and retained proteins were analyzed by SDS-PAGE and Coomassie brilliant blue staining. (B) Quantitation of the binding between candidate effectors and SUMO2. The binding affinity was calculated from the band intensity of SUMO2 to GST-effectors. The relative binding affinity was calculated by dividing the binding affinity of each GST-effector by the binding affinity of GST-SulF. Data were collected from three independent experiments and shown is the mean ± s.e. (C) Immunoprecipitation showing the binding of SUMOylated Rab7 and SulF. HEK293T cells were transfected to express Flag-Rab7 and HA-SUMO2 for 36 h. Beads coated with Flag-SUMO-Rab7 were incubated with recombinant effectors for 1 h and the retained proteins were detected by immunoblotting with appropriate antibodies. (D-F) Deletion of sulF from strain Lp02rpsLK88R did not impair its intracellular replication in BMDMs. The indicated bacterial strains were used to infect BMDMs, the protein level of SulF (D), the ratio of large LCVs (E) and total bacterial counts for a 72-h experimental duration at 24-h intervals (F) were analyzed. In all cases, quantitative results (mean ± s.e.) were from three independent experiments each. (TIF) [file ppat.1011783.s006.tif]

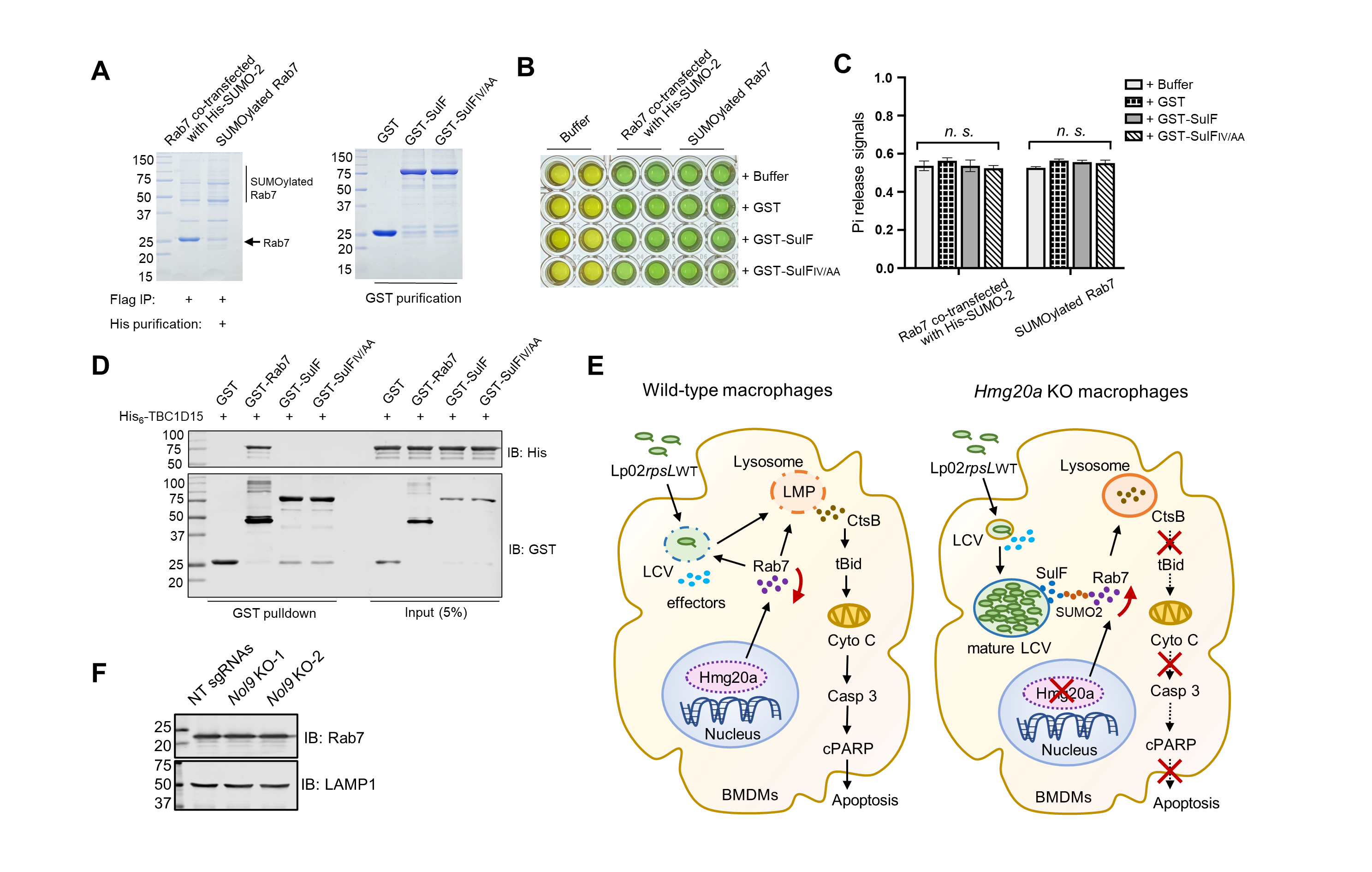

Supplement: S7 Fig — (A) SUMO-Rab7 obtained by a two-step purification procedure was evaluated by SDS-PAGE and CBB staining. (B-C) SulF does not impact the GTPase activity of Rab7. Rab7 or SUMOylated Rab7 were incubated with SulF for 30 min. Then reactions were stopped and released Pi signals at 488nm were recorded using a plate reader. The image of one experiment (B) and the activity measured by released free phosphate from three independent experiments mean ± s.e. (C) were shown. (D) SulF did not interact with TBC1D15. GST-Rab7 or GST-SulF (or its SIM-defective mutant) was mixed with His6-TBC1D15 and the potential protein complexes were captured with GST beads. Retained proteins separated by SDS-PAGE were detected by immunoblotting with the indicated antibodies. (E) Summary model for SulF-mediated recruitment of SUMOylated Rab7 in Hmg20a knockout macrophages. (F) Nol9 knockout did not affect Rab7 expression. Proteins from the indicated cells separated by SDS-PAGE were probed with antibodies specific for Rab7 and LAMP1, respectively. (TIF) [file ppat.1011783.s007.tif]
