# Supplementary material for: Legionella pneumophila exploits the endo-lysosomal network for phagosome biogenesis by co-opting SUMOylated Rab7
Source: PLoS Pathog. 2024 May 13;20(5):e1011783. doi: 10.1371/journal.ppat.1011783 (PMC11115209; doi:10.1371/journal.ppat.1011783)
Supplement: Attachment — Submitted filename: Response to review (PPATHOGENS-D-23-01887) clean.docx [file ppat.1011783.s008.docx]

We wish to thank the three expert reviewers for their time and efforts in evaluating our manuscript. Their insightful and constructive comments and suggestions have allowed us to more carefully evaluate our results, think more deeply about some of the key experiments. We have revised the manuscript in accordance with their comments and suggestions. Our point-to-point response to these comments is as follows:

**Part I - Summary**

Please use this section to discuss strengths/weaknesses of study, novelty/significance, general execution and scholarship.

**Reviewer #1**: The manuscript by Li et al. explores the significance of the human small GTPase Rab7 in the infection process by the bacterium Legionella pneumophila.

Legionella infects eukaryotic organisms, being taken up by phagocytosis of macrophages. During infection, Legionella secretes bacterial effectors that manipulate the host cell. A characteristic of this process is the formation of the Legionella-containing vacuole (LCV), where the bacteria can multiply.

In their work, the authors investigate why some Legionella strains cannot survive in bone marrow-derived macrophages (BMDM). Through genetic manipulation of BMDM, they identify a reduced presence of the human small GTPase Rab7 as the cause of decreased viability. Based on mutagenetic analysis, the authors speculate that the covalent modification of Rab7 with the protein SUMO is crucial for the successful establishment of Legionella infection. This speculation is supported by the characterization of bacterial effectors carrying specific SUMO-binding properties. One of these effectors, SulF, binds to SUMO-modified Rab7 and appears to keep it in an active state, where the enzymatically stimulated GTP hydrolysis of Rab7 is inhibited.

This work presents an astonishing discovery process, starting from an infection biology observation to the identification of a cellular mechanism and the responsible Legionella effector SulF. However, the paper largely focuses on outlining the pathway and places less emphasis on the detailed analysis of the molecular mechanisms of Rab7 manipulation by SulF. As a result, the actual knowledge gain for the Legionella infection process is limited, leading me to believe that publication in a more specialized journal would be more appropriate.

**Response:** We appreciated the positive comments by the reviewer. We believe that our somewhat twisted path for the discovery of the potential co-option lysosomal proteins such as Rab7 has significantly expanded our appreciation of the role of this cellular process in the biogenesis of the Legionella-containing vacuole (LCV), particularly for *bona fide* wild-type strains of this pathogen (please keep in mind that those commonly used laboratory “wild-type” strain such as Lp02 and JR32 harbor a K88R mutation in their *rpsL* gene). Our finding that SulF functions to recruit Rab7 to the LCV also provides a molecular explanation for the association of Rab7 with the bacterial phagosome described by Dr. Horwitz and colleagues more than two decades ago (*Infection Immunity 2000*^1^). Importantly, the *bona fide* wild-type Philadelphia 1 (no mutations in the *rpsL* gene) was used in the study by Dr. Horwitz’s group. The findings detailed in our submission have provided significant new insights into our understanding of the interactions between *L. pneumophila* and macrophages, and are well within the scope of *PLoS Pathogens*.

**Reviewer #2:** This is a comprehensive and exceptional study that identifies the molecular mechanisms by which certain strains of Legionella pneumophila (Lp) expressing a wild-type version of the ribosomal protein, RpsL, induce rapid cell death in mouse macrophages and thereby restricting Lp replication. Previously, the Luo lab found that a K88R mutation in RpsL conferring streptomycin resistance also attenuated the cell death response whereas a K43N mutation had no impact on attenuating cell death but still conferred streptomycin resistance. Here the authors performed a CRISPR screen that showed cell death was influenced by Hmg20a, a host factor that regulates lysosomal damage and an unrelated nucleolar protein Nol9 that possesses polynucleotide 5'-kinase activity. The authors go on the show that Hmg20a depletion rescues mouse macrophages from Lp induced cell death, as does Rab7 overexpression, and they discover a Lp effector, SulF that recruites SUMOylated Rab7 to the Legionella containing vacuole. Although the phenotype is restricted to mouse macrophages, this study has relevance to antibiotic resistance mechanisms and where such mutations intersect with and influence host-pathogen interactions.

**Response:** We are thankful for the very positive remarks from the reviewer on our work.

**Reviewer #3**: The authors build upon their previous study showing that Legionella pneumophila strains harboring a WT rpsL gene induce lysosomal damage and apoptosis and cannot replicate in mouse macrophages. Through a CRISPR/Cas9 screen, they identify the host genes Hmg20a and Nol9 as being required for restricting Lp carrying WT rpsL. They focus on Hmg20a for the remainder of the manuscript and find that Hmg20a represses the expression of Rab7 and other endolysosomal proteins, and that overexpression of Rab7 rescues the ability of Lp carrying WT rpsL to replicate in macrophages. Furthermore, they find that the Dot/Icm effector SulF recruits SUMOylated Rab7 to the bacterial phagosome. Their data indicate that SulF inhibits Rab7 GTPase activity by blocking binding of the GAP TB1D15.

The studies are well executed and the manuscript tells a convoluted but interesting story. The authors’ study of the Lp02rpsLWT model enabled them to obtain new insight into the activity of a Lp effector, SulF, in recruiting Rab7 to the LCV. Furthermore, the manuscript indicates a role for Rab7 in regulating intracellular replication of Lp02rpsLWT BMDMs. Overall, their data suggest that Lp interacts with the endolysosomal network during LCV biogenesis in macrophages. However, some of the conclusions are based on findings where the authors are examining SulF overexpression or deficiency in the Lp02rpsLWT strain, often in macrophages overexpressing Rab7. I would like to see the authors perform some of the experiments in macrophages that are not overexpressing Rab7, and examine Rab7 overexpression or SulF deficiency or overexpression in the context of the Lp02rpsLK88R strain, in addition to some of my other suggestions listed below.

**Response:** We wish to thank the reviewer for her/his favorable comments about our work and the suggestion for additional experiments to clarify a few points or to strengthen the conclusions. We have performed these experiments. Please refer to our responses to your specific comments.

**Part II – Major Issues:** Key Experiments Required for Acceptance

Please use this section to detail the key new experiments or modifications of existing experiments that should be absolutely required to validate study conclusions.

Generally, there should be no more than 3 such required experiments or major modifications for a "Major Revision" recommendation. If more than 3 experiments are necessary to validate the study conclusions, then you are encouraged to recommend "Reject".

**Reviewer #1**: I would like to outline my main criticisms below:

- The detailed presentation of the discovery process regarding the importance of Rab7 for infection is extensive. While it is understandable that a significant portion of the research effort is dedicated to this aspect, the exposition of the discovery process seems to contribute minimally to knowledge gain. The complexity of this detailed process significantly impacts the understanding of the work. Substantially shortening this aspect or moving it to the supplementary materials would likely enhance the overall comprehension of the study. The main focus should be on the molecular mechanisms of Rab7 manipulation by SulF.

**Response:** We’d like to thank the reviewer for raising this point, which to us was also a balance somewhat difficult to strike in the preparation of the manuscript. The known biochemical activity of Hmg20a suggests that this protein did not directly impact the biogenesis of the LCV, which prompted to take the long route to discover the role of lysosomal proteins such as Rab7 in this process. In the end, we inclined to include the description of the logics we used to justify our experiments, which in our mind will allow readers to better understand the results. In the revision, we have shortened this portion of text to more concisely present our findings.

- An important finding is the discovery that SulF appears to suppress GAP-mediated GTP hydrolysis of Rab7. However, the presented data raise concerns. Are the protein concentrations in the "GTPase activity assay" chapter (line 657) correctly stated? The concentrations of Rab7 (1mM), SulF (0.1mM), and TBC1D15 (0.1mM) seem unusually high. Could the authors possibly mean uM instead of mM? Furthermore, the inhibitory effect of SulF on Rab7 GTP hydrolysis is not explainable under the given protein concentration conditions. Since the concentration of Rab7 is 10 times higher than that of SulF and TBC1D15, 90% of Rab7 should be accessible to TBC1D15 at all times, and the GTP hydrolysis should hardly be affected. The observed effect could only be explained if SulF directly prevents the binding of the GAP by binding to TBC1D15. However, this has not been demonstrated. How do the authors explain this effect?

**Response:** We’d like to thank the reviewer for pointing out this error. These are indeed typos (to enter “μ"**,** we often enter it as “m” then convert “m” into “μ" by changing it to symbol. Here we did not finish the conversion). Nevertheless, we have redone the experiments and the correct protein concentrations used are as follows: Rab7 (0.1μM), SulF (0.1μM), and TBC1D15 (0.1μM). The molar ratio among SUMOylated Rab7, SulF and TBC1D15 is 1:1:1 in our previous experiments. In addition, we have examined the impact of TBC1D15 on GTP hydrolysis of Rab7 in reactions with different ratios of SulF or TBC1D15 to Rab7. Our results indicate that SulF inhibits it’s the GTPase activity of Rab7 in a dose-dependent manner **(Fig 7D-7G)**.

- The SUMOylation position (K175) of Rab7 is located far from the usual binding sites for regulatory proteins and other interaction partners. Therefore, it is difficult to conceive how the SUMO-mediated binding of SulF to Rab7 could have a competitive effect on GAB binding (TBC1D15). Even though a detailed analysis of the molecular mechanisms of SulF binding might be beyond the scope of this study, a thorough discussion of this issue would be appropriate. Could the authors speculate on whether SulF has direct or indirect effects on the action of TBC1D15?

**Response:** Thank you for this very insightful point. Although the SUMOylation site K175 is distal to regions of Rab7 commonly known to be involved in protein-protein interactions, the binding of SulF, a protein of approximately 63 kDa (546 amino acids) likely will induce conformational changes in Rab7 or the (bulky) SulF protein may directly block the access by interacting proteins such as the GAP TBC1D15. We have added this discussion in the revised manuscript **(Line 478-482)**.

**Reviewer #2**: I have no suggestions for further experiments as the study is comprehensive and large covering multiple techniques that have been performed and presented to a high standard. I have some minor suggestions to explain the purpose of the manuscript for readers who are not familiar with the original study.

1. Line 78, RpsL here needs more context of the original findings, intersection with Strep resistance and what RpsL does.

**Response:** We thank the reviewer for raising this point, we have added more background information about RpsL and Strep resistance in the revised manuscript **(Line 88-94)**.

2. Please add statistical analyses to all graphs, eg. Fig 1A, 1H etc, if not significant please say so and in the figure legends, state the statistical test used.

**Response:** We appreciate the careful reading of our manuscript by the reviewer; we have added statistical analyses to all graphs in the revised manuscript.

3. The discussion should include some thoughts on why the mutant K88R does not trigger lysosomal induced cell death

**Response:** The detailed mechanism underlying this phenotype is still under investigation. We have obtained preliminary results suggesting that the K88R mutation in RpsL alters the abundancy of a number of *L. pneumophila* proteins, including some Dot/Icm substrates. A simple model is the K88R mutation lowered the abundancy of one or more proteins capable of inducing lysosomal cell death in mouse BMDMs. We have added a short discussion of this speculation in the revised text **(Line 458-463)**.

4. The discussion should also compare the results with human cells, ie. why is the mechanism found in BMDM.

**Response:** Thank you for another important point. Indeed, we have speculated the possible mechanisms for such sharp contrast between human macrophages and mouse BMDMs in our earlier publications (*PLoS Pathogens* 2015^2^, *J Nat Sci.* 2015^3^). Our hypothesis is that there is one or more mouse-specific receptors that function to detect (protein) ligands secreted (more abundantly) by RpsL wild-type strains. We have included a brief discussion of this important point in the revised manuscript **(Line 463-470)**.

5. I think the intersection of antibiotic resistance mechanisms and impact on virulence could also be mentioned as this is exemplified here.

**Response:** Thank you! We have added a brief discussion on the relationship between antibiotic resistance and virulence in the revised text **(Line 458-463)**.

**Reviewer #3**: Major comments:

1. The authors show that overexpression of active Rab7 enables Lp02rpsLWT to replicate in BMDMs. The authors should test if Rab7 overexpression also leads to reduced cell death in BMDMs infected with Lp02rpsLWT, as they showed for BMDMs lacking Hmg20a.

**Response:** We have followed the suggestion to perform this experiment, our results indicate that overexpression of Rab7 reduced cell death in BMDMs induced by infection with Lp02*rpsL*_WT_ strain **(Fig S4A-S4C)**.

2. Is the phenotype that Rab7 overexpression supports increased LCV formation and intracellular replication of Lp02rpsLWT specific to this strain? Or does it also extend to the Lp02rpsLK88R strain?

**Response:** Thank you! We have examined this point and our results indicate that overexpression of Rab7 did not detectably impact intracellular growth of strain Lp02*rpsL*_K88R_ **(Fig S4D-S4F).**

3. In figure 5, the authors examine large LCV formation and intracellular replication of a Lp02rpsLWT mutant lacking sulF in macrophages overexpressing Rab7, and they find that this Lp02 mutant has a defect in intracellular replication. This is an interesting finding, but it is in the context of Rab7 overexpression. The authors should also examine the phenotype of Lp02rpsLWT mutant lacking sulF as well as the Lp02rpsLK88R mutant lacking sulF in WT macrophages that are not overexpressing Rab7.

**Response:** We thank the reviewer for raising this point. Results from our new experiments suggest that deletion of *sulF* from strain Lp02*rpsL*_K88R_ did not impair its intracellular replication in BMDMs **(Fig S6E and S6F)**.

4. The authors’ data indicate that Rab7 is more robustly recruited to the vacuoles of Lp02rpsLWT than Lp02rpsLK88R, as LP02rpsLWT expressed higher levels of SulF than Lp02rpsLK88R and overexpression of SulF in Lp02rpsLK88R led to increased Rab7 recruitment to the LCV. The authors examine the ratio of large LCVs and intracellular replication in Lp02rpsLWT overexpressing SulF, but not Lp02rpsLK88R overexpressing SulF. The authors should also perform experiments to examine the phenotype of Lp02rpsLK88R overexpressing SulF in terms of large LCV formation and intracellular replication. This would help ascertain whether increased SulF expression is responsible for the Lp02rpsLWT phenotype in terms of decreased intracellular replication in WT BMDMs.

**Response:** We thank the reviewer for raising this point. We have performed this experiment and the results show that overexpression of SulF did not detectably impact the intracellular growth of stain Lp02*rpsL*_K88R_ in BMDMs **(Fig 6K-6M)**.

5. Based in part on the data shown in figure 7D, the authors make the conclusion that SulF interferes with the binding of SUMOylated Rab7 to TBC1D15. However, the difference in intensity of TBC1D15 bands between conditions with and without SulF is quite subtle. It would be helpful if the authors quantitated the TBC1D15 band densities and normalized to the input bands across several experiments.

**Response**: Thank you! We have included statistical analyses of the intensity of relevant protein bands from multiple independent experiments **(Fig 7E)**.

**Part III – Minor Issues:** Editorial and Data Presentation Modifications

Please use this section for editorial suggestions as well as relatively minor modifications of existing data that would enhance clarity.

Reviewer #1: (No Response)

Reviewer #2: None

Reviewer #3:

1.The manuscript abstract’s second sentence is “The mechanism of this unique infection-induced cell death remains unknown,” which leads the reader to think that the authors will identify the mechanism in this manuscript. However, although the authors identified a role for Hmg20a and Rab7, the precise mechanism underlying Lp02rpsLWT-induced cell death is still unknown. Thus, I would suggest that the authors remove this sentence, as it is somewhat misleading.

**Response:** We agreed with this point and have revised this sentence to make the description more consistent with our findings **(Line 26-29)**.

2. Line 186: typo- “BMMDs” should be “BMDMs”

**Response:** Thanks! This typo has been corrected.

3. Line 459: typo- “C56BL/6” should be “C57BL/6”

**Response:** Thanks! This typo has been corrected.

PLOS authors have the option to publish the peer review history of their article (what does this mean?). If published, this will include your full peer review and any attached files.

If you choose “no”, your identity will remain anonymous but your review may still be made public.

Do you want your identity to be public for this peer review? For information about this choice, including consent withdrawal, please see our Privacy Policy.

Reviewer #1: No

Reviewer #2: No

Reviewer #3: No

**References:**

1.	Clemens DL, Lee BY, Horwitz MA. Mycobacterium tuberculosis and Legionella pneumophila Phagosomes Exhibit Arrested Maturation despite Acquisition of Rab7. *Infection and Immunity*. 2000;68(9):5154-5166. doi:10.1128/iai.68.9.5154-5166.2000

2.	Zhu W, Tao L, Quick ML, Joyce JA, Qu JM, Luo ZQ. Sensing cytosolic RpsL by macrophages induces lysosomal cell death and termination of bacterial infection. *PLoS Pathog*. 2015;11(3):e1004704. doi:10.1371/journal.ppat.1004704

3.	Zhu W, Luo ZQ. A new way to detect the danger: Lysosomal cell death induced by a bacterial ribosomal protein. *J Nat Sci*. 2015;1(6).
